# Towards Realistic 3D Models of Tumor Vascular Networks

**DOI:** 10.3390/cancers15225352

**Published:** 2023-11-09

**Authors:** Max C. Lindemann, Lukas Glänzer, Anjali A. Roeth, Thomas Schmitz-Rode, Ioana Slabu

**Affiliations:** 1 Institute of Applied Medical Engineering, Helmholtz Institute, Medical Faculty, RWTH Aachen University, Pauwelsstraße 20, 52074 Aachen, Germanyglaenzer@ame.rwth-aachen.de (L.G.); smiro@ame.rwth-aachen.de (T.S.-R.); 2 Department of General, Visceral and Transplant Surgery, RWTH Aachen University Hospital, Pauwelsstrasse 30, 52074 Aachen, Germany; 3 Department of Surgery, Maastricht University, P. Debyelaan 25, 6229 HX Maastricht, The Netherlands

**Keywords:** image registration, segmentation, vascular network model, tumor, reconstruction, image processing, histologic images

## Abstract

**Simple Summary:**

Three-dimensional models of tumor vascular networks are of significant importance for in vitro and in silico investigations of, for example, the efficiency of anti-cancer drugs in an early stage of clinical transition and can be potentially used for the development of in vitro systems as 3D-printable vascular networks to facilitate personalized medicine and randomized controlled clinical trials. In this work, histologic slices of a human pancreatic tumor are used as examples to establish an algorithm-based method that enables the reconstruction of a 3D vascular network model. The advantages of this method are high resolution and accuracy concerning the characteristics of the vascular network (e.g., density, trajectory of vessels).

**Abstract:**

For reliable in silico or in vitro investigations in, for example, biosensing and drug delivery applications, accurate models of tumor vascular networks down to the capillary size are essential. Compared to images acquired with conventional medical imaging techniques, digitalized histological tumor slices have a higher resolution, enabling the delineation of capillaries. Volume rendering procedures can then be used to generate a 3D model. However, the preparation of such slices leads to misalignments in relative slice orientation between consecutive slices. Thus, image registration algorithms are necessary to re-align the slices. Here, we present an algorithm for the registration and reconstruction of a vascular network from histologic slices applied to 169 tumor slices. The registration includes two steps. First, consecutive images are incrementally pre-aligned using feature- and area-based transformations. Second, using the previous transformations, parallel registration for all images is enabled. Combining intensity- and color-based thresholds along with heuristic analysis, vascular structures are segmented. A 3D interpolation technique is used for volume rendering. This results in a 3D vascular network with approximately 400–450 vessels with diameters down to 25–30 µm. A delineation of vessel structures with close distance was limited in areas of high structural density. Improvement can be achieved by using images with higher resolution and or machine learning techniques.

## 1. Introduction

Three-dimensional models of vascular networks have the potential to improve the accuracy of computational and experimental investigations in many biomedical fields. Some of these are biosensing, organ-on-a-chip (OOAC), tissue engineering and scaffolding, and development of anti-cancer drugs (ACDs) in cancer therapy. In biosensing, vasculature models are necessary for evaluation of the sensors’ efficiency both in silico [1] and in vitro [2]. However, accurate vasculature models would increase the reliability of these results. Similarly, for OOACs, microfluidic systems are built to simulate the physiology of human organs [3]. Current OOACs allow for the use of 3D structures [4] for which, in general, vascularization is still needed and part of ongoing research [5]. For the development of ACDs, accurate tumor models are needed to evaluate their effectiveness in early-stage development [6]. The ACDs’ development also includes approaches such as magnetic drug targeting to realize more efficient drug delivery [7,8]. This approach can be combined with other tumor therapies to increase their effectiveness [9,10,11,12,13].

Animal models deliver the necessary tissue function but also have limitations (i.e., the comparatively small number of specimens that can be investigated and high variability in the experimental conditions, e.g., due to biodynamic signatures) [14]. In addition, it is recommended to avoid animal experiments whenever possible [15]; however, reliable alternatives exist only for macroscopic structures [16] that do not mimic human tissue function. In tissue engineering, 3D models of vascular networks are already used for the design of scaffolds [17]. The models are usually based on artificially generated data [18] or on medical imaging data (e.g., from micro-computed tomography (µCT)) [19]. The artificially generated models include approaches that allow reverse-engineering; however, they still need rigorous experimental validation. Methods such as CT, µCT, and two-photon microscopy allow imaging of vasculature without destroying the tissue, and spatial encoding is available. As these methods are well established, the model acquisition is relatively fast. Furthermore, these methods allow imaging in vivo. Explantation of the tumor is not necessary. However, the resolution of CT and µCT images is too low (CT: approx. 1 mm, µCT: approx. 5 µm) to sufficiently delineate capillaries [19,20,21]. For two-photon microscopy, the resolution is around 100 nm [22], but the tissue penetration depth is comparatively low (approx. 1 mm). This method was also used for the investigation of in vivo drug delivery [23].

In clinical practice, reliable treatment planning is of paramount importance. Hence, to further clinical translation of the above-mentioned approaches, accurate in silico investigation based on realistic vascular network models plays a key role. In this work, histologic slices of a human pancreatic tumor are used as examples to establish a new approach of building a virtual 3D vascular network model. The advantages of this method compared to the ones mentioned above are high resolution, accuracy concerning the characteristics of the vascular network (e.g., density, trajectory of vessels), and a potential use for in vitro systems as 3D-printable vascular networks to facilitate personalized medicine and randomized controlled clinical trials. Such 3D networks can enable precise simulations of blood flow dynamics, aiding in the development of targeted treatments and predicting patient-specific responses.

**Figure 1:**
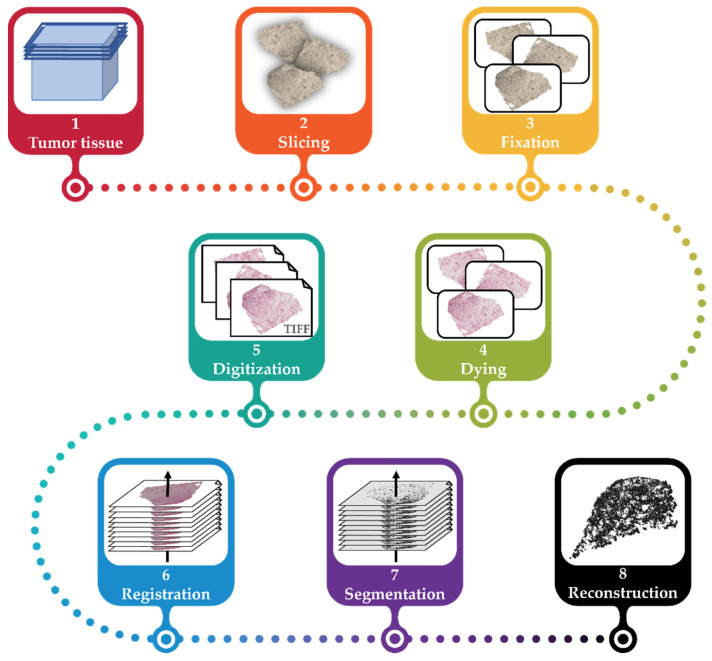
The general workflow for the reconstruction of the vascular network. The tumor tissue is explanted and embedded in paraffin wax (1), sliced into 2.5 µm slices (2), fixated in 3.5% formaldehyde, and placed on slides (3). The tissue on each slide is then immunohistochemically stained (4) and then digitized (5). The resulting images are registered (6). Using segmentation, vascular structures are extracted (7) and reconstructed for the vascular model of the initial tumor (8).

This workflow for the generation of a vascular network model of a tumor using histological images presented in this work consists of several steps: slicing a tumor, fixation of slices on slides, staining the structures of interest, digitization of slides, registration of the digitalized slides (i.e., alignment to the same coordinate system), image segmentation of the structures of interest (i.e., vessels), and reconstruction of the registered and segmented images to a 3D model (cf. Figure 1).

The major challenges for the generation of a 3D model from histologic images compared to state-of-the-art procedures are the registration of high-resolution and highly repetitive features, segmentation robust to artifacts from sample preparation, and implementation that allows for upscaling of the number of images used. To overcome these challenges, this work uses a combination of area- and feature-based registration, and segmentation including intensity- and color-based thresholds and heuristic analysis. Here, we present as an example the registration and reconstruction of a vascular network from histologic slices applied to 169 tumor slices. The algorithms are discussed, and results are compared to state-of-the-art registration (see Table 2), segmentation, and reconstruction approaches in Section 3.

## 2. Materials and Methods

The general workflow for the generation of the 3D vascular network is shown in Figure 1. Steps (1) to (5) are described in Section 2.1, step (6) is described in Section 2.2 and Section 2.3, step (7) in Section 2.4, and step (8) in Section 2.5.

### 2.1. Sample Preparation

**Figure 2:**
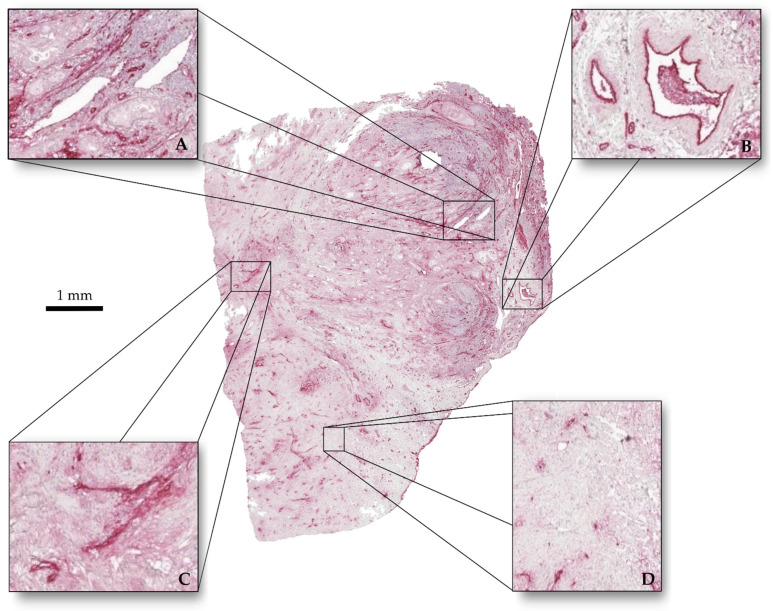
Tumor slice immunohistochemically stained. (**A**) Vessels with clear outlines and homogeneous interiors. (**B**) Vessels with clear outline and perturbed interiors. (**C**) Vessels with perturbed outlines and no interiors. (**D**) Stained cell nuclei.

After informed consent of the patient and with permission of the local IRB (EK206/09), a piece of a human pancreatic ductal adenocarcinoma was used after resection. It was fixed in a 3.5% formaldehyde solution (Otto-Fischar GmbH, Saarbrücken, Germany). The tumor was embedded in paraffin wax and cut in 169 slices of 2.5 µm thickness each using a microtome (HM 340 E, Thermo Fisher Scientific Inc., Waltham, MA, USA). Slices were manually placed on slides (Starforst, Engelbrecht Medizin- und Labortechnik, Edermünde, Germany) and dried overnight at 56 °C. The slices were immunohistochemically stained (Figure 2) with the antibody “von Willebrandt-factor” (DakoCytomation, Glostrup, Denmark) using the ZytoChem Plus AP Polymer System (Mouse/Rabbit) (Zytomed Systems, Berlin, Germany) and Hematoxylin (Zytomed Systems, Berlin, Germany). Then, the slides were covered with Vitro-Clud (R. Langenbrinck GmbH, Emmendingen, Germany).

The slides were digitalized using a microscope (Axio Imager.Z2, Carl Zeiss Microscopy Deutschland GmbH, Jena, Germany). The slide images were converted and cropped to a size of 13020 × 10422 px², resulting in images with a pixel size of 0.5 µm.

### 2.2. Preprocessing

Prior to the registration, the images were preprocessed to achieve better registration results. First, the background of the images (i.e., the area of the image not showing any tissue) was replaced with white. This is necessary, as the registration algorithm (see Section 2.3) requires a white background. Additionally, a white margin (12.5% of the image size) was added to each image. This made the image registration more robust against losing image information through rotation (cf. Section 2.3 on registration). Second, the imregtform function of Matlab (The MathWorks, Inc., Natick, MA, USA) was used to perform a so-called similarity transformation between consecutive images. This similarity transformation included rotation, translation, reflection, and scaling of the images. However, it did not include elastic transformation, which was performed in a later step (see Section 2.3).

One image preprocessing took only few seconds and could be computed in parallel.

All computations were performed with computing resources granted by RWTH Aachen University. The RWTH Aachen University High-Performance Cluster provides access to computational nodes using 2 Intel Xeon Platinum 8160 Processors “SkyLake” with 2.1 GHz, 24 cores each. Each node hast 48 cores and 192 GB of main memory.

### 2.3. Registration

Because of the slides’ preparation (see chapter 2.1), a misalignment of the images was inevitable. Therefore, an image registration was required, which transfers the images into the same coordinate system. For the registration, the open-source software package Fiji [24] was used.

The registration algorithm presented here is based on the works of Wang et al. [25,26], which was available as a plug-in for Fiji (version 1.45) called ImproveCWR. We updated the bUnwarpJ version 3 [27,28] within this plugin in order to process images with larger file sizes. We also adjusted the ImproveCWR plugin to be able to save the transformation parameters of each registration.

**Figure 3:**
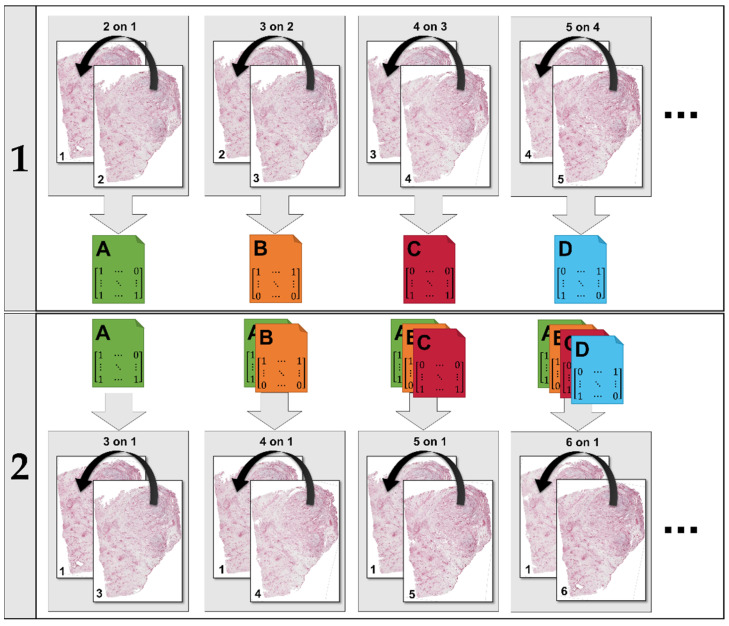
The registration proceeds in two steps. In the first step, each image is aligned to the previous one (i.e., 2 to 1, 3 to 2, etc.). Each of these registrations yield a transformation that can be saved to file (A, B, etc.). In the second step, each image is aligned to the first one in the set using concatenations of the transformations saved in the first step. All calculations within a step are performed in parallel.

As registering one image after the other would have been very time consuming when working with large numbers of images, we chose a two-step process that parallelized the registration, as described in Figure 3.

The adjusted ImproveCWR registration algorithm was validated using the training datasets of the ANHIR challenge [29,30]. This provides 230 histological image pairs with annotated image features (i.e., SIFT features). Using these registered images, the relative target registration error (*rTRE*) for every annotated image feature *l* in every image pair (*i*,*j*) was calculated as follows:

(1) $${rTRE}_{l}^{ij}=\frac{{\left\|{\hat{x}}_{l}^{j}-x_{l}^{i}\right\|}_{2}}{d_{j}}$$

with feature position *x*, the transformed feature position $\hat{x}$, the diagonal of the image *d_j_*, and the Euclidian norm ${\left\|\cdot \right\|}_{2}$. Then, the median value of all features in a pair of images and the average of all medians were calculated.

The robustness *R_i_* is the number of improved features compared to the total number of features in the set *L_i_* in the image *i*. To calculate *R_i_*, the relative initial registration error (*rIRE*) was determined as follows:

(2) $${rIRE}_{l}^{ij}=\frac{{\left\|x_{l}^{j}-x_{l}^{i}\right\|}_{2}}{d_{j}}.$$

Using *rTRE* and *rIRE*, *R_i_* can be calculated:

(3) $$R_{i}=\frac{1}{\left\|L_{i}\right\|}\sum_{l\in L_{i}}k_{l},\;\;\;\;k_{l}=\left\{\begin{array}{cc} 1, & {rTRE}_{l}^{ij}<{rIRE}_{l}^{ij}\\ 0, & {rTRE}_{l}^{ij}\geq {rIRE}_{l}^{ij} \end{array}\right.$$

The average *R_i_* over all images was calculated.

As the dataset of the ANHIR challenge includes a variety of tissues, we created a subset of images including only images that are similar to the tissue used in this work and performed a separate validation as described above (Equations (1)–(3)). We calculated the average *R_i_* and average median *rTRE* for the subset of 90 image pairs and the complete set of 230 image pairs.

### 2.4. Segmentation

The images were converted to grayscale as the segmentation algorithm proposed in this section only relies on the pixel intensities. The brightness for each image was homogenized using the contrast-limited adaptive histogram equalization (CLAHE) algorithm [31]. Then, a median filter was used to reduce noise (from the cell nuclei).

**Figure 4:**
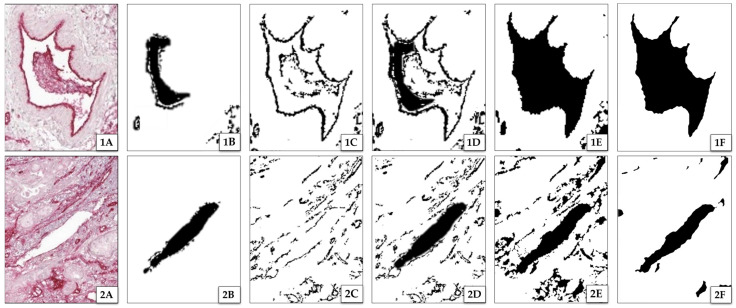
Results of segmentation steps for two image excerpts 1 and 2. (**A**) shows excerpts with one vascular structure each. (**1A**) has a well-defined contour with a perturbed interior, whereas (**2A**) has a clear interior with a perturbed contour. (**B**) shows the results of extracted interiors of vessels, and (**C**) shows the results of their extracted contours. (**D**) is a fusion of (**B**) and (**C**), indicating significant differences in possibly detected interiors and contours (white spaces between interior and contours). (**E**) shows the result of the segmentation after dilating and filling the detected vessels to their contours (see step (v)). Images (**F**) show the final segmentation results with applied smoothing.

Starting from here, the segmentation was performed as described in Figure 4, consisting of five steps: (i) delineation of vessel interiors, (ii) delineation of vessel contours, (iii) removal of unwanted structures, (iv) fusing contour and interior information, and (v) filling of remaining gaps.

**Figure 5:**
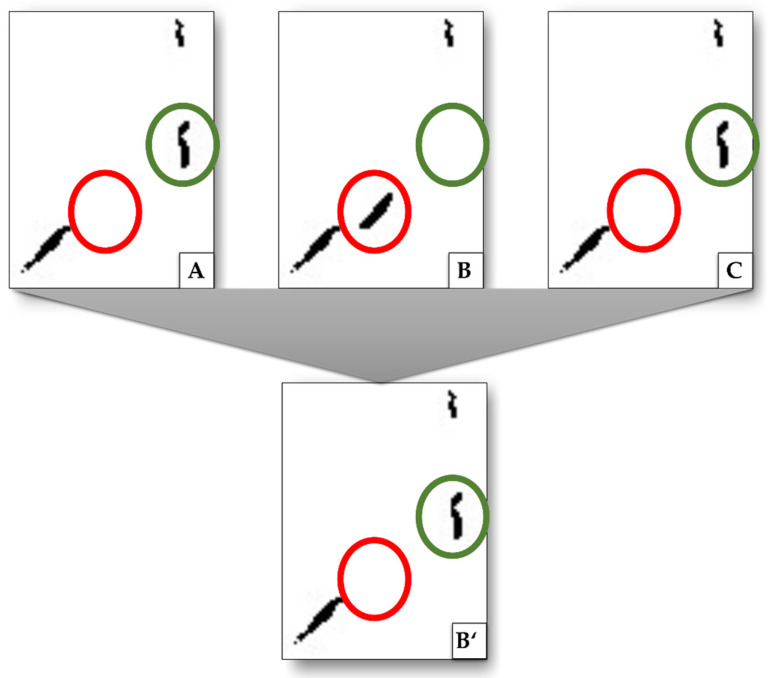
Exemplary procedure to remove structures that are related to damage artifacts and not to vessels as well as to add missing structures by interpolation. Images (**A**–**C**) show excerpts from consecutive images, (**B’**) is the result after (**B**) is compared to its next neighbors. Highlighted in green is a structure detected in image (**A**) and (**C**), but not in (**B**). By interpolation, this structure is added to image (**B’**). Highlighted in red is a structure that only appears in (**B**) but not in (**A**) or (**C**). This is interpreted as damage artifacts and is removed (does not appear in (**B’**)).

(i) The first step of the segmentation process was the delineation of vessel interiors (see Figure 4B, black area). These are prominent due to their bright and plain white appearance. To isolate these areas, a threshold with a cut-off value of 82.35% color intensity is used. This threshold was determined using Otsu’s method [32]. Because of the preprocessing, one cut-off value was sufficient for all images. Figure 4(1B) shows that the lumen was only partially delineated due to presence of other structures, such as cells or debris, within the vessel. However, these missed parts of the lumen will be gained by reconstruction (see below step (iii)).
(ii) The second step was the delineation of the contours of the vessels in the preprocessed image (see Figure 4C). This includes the contour of vessel interiors (delineated in step one) and of collapsed vessels that do not have an interior. Because of the staining, the latter are darker compared to the surrounding tissue. Hence, the intensity of the vessel contours varies throughout the image. As the segmentation is based on thresholding, the image was rasterized and a local cut-off value $I_{cut-off}$ was calculated for every rasterized part as follows:

(4) $$I_{cut-off}=\mu +x\cdot \sigma \;,$$

where μ is the mean intensity, σ is the standard deviation, and x is an empirical value. The results of both segmentation steps were binarized. In this way, two types of images were created: one with delineated vessel interiors and the other with delineated vessel walls (contours). In both image types, damage artifacts that occurred during the slicing process and real vessel structures were not differentiated (for example, there was no difference between delineated fissures and vessel interiors).

(iii) In the third step, unwanted structures (such as cell nuclei, megakaryocytes, or damaged tissue) were removed from the images that resulted after processing according the two previous steps. This was performed as follows:
  1. Pixels that were not related to vessels (bright or dark spots in the image) were removed by application of a sliding window algorithm. This algorithm removes every structure that completely fits into a region of the image with a size of n×n pixels, where n is an experimental value. If n is too big, vessel structures are deleted. If n is too small, the unrelated pixels might not be removed effectively. The size of n depends on the $I_{cut-off}$ and the magnification used.
  2. Structures in the image of damaged tissue (induced by the manual preparation of the slides) are removed by using the Moore–Neighbor tracing algorithm [33] with Jacob’s stopping criterion [34]. Usually, after segmentation, vessel interiors (delineated in step (i), Figure 4B) show continuous contours of intensely stained tissue (delineated in step (ii), Figure 4C). These contours are dilated by five pixels. Only vessel interiors that have at least 45% of a corresponding contour length are kept.
  3. Further removal of damaged tissue was performed by comparing vessel interiors of three consecutive images using the Moore–Neighbor tracing algorithm. Repeating structures in the images were kept, the other ones were discarded (Figure 5, red circles). Further, interiors not found in step (i) were added when they appeared in two of three images (Figure 5, green circles). To increase accuracy, these removal and addition procedures can be extended to a higher number of consecutive images that are analyzed. For both removed and added structures, a small offset up to 10 pixels of the position of the structure between two consecutive images is allowed to compensate for errors in the registration.

(iv) The fourth step of the segmentation process was the fusion of images with the information on vessel contours with those of the vessel interiors (see Figure 4D). The interiors are a bit smaller than the contours, due to different cut-off values (cf. steps (i) and (ii)) in the respective segmentations.
(v) The fifth step was the closing of open ends of contours to form closed contours and filling of the gaps between these closed contours and vessel interiors (see Figure 4E). The algorithm uses binary dilation [35] with a disk of radius 5 px on every pixel. If any other pixel is within the radius, the gap is closed. Filled structures were discarded if the filled area was larger than the area of the biggest vessel in the respective image. This procedure was repeated, and the maximum gap-size was doubled for each iteration. If a filling was discarded for the first time, the disc size of the last iteration was increased by 5 px for every following iteration when the filling was discarded for the second time.

For further filling of remaining gaps, contours were dilated, then filled and eroded afterwards. To prevent clustering in areas of high vessel density, the image was rasterized, and the dilation was adapted to the current raster’s vessel density.

Further refinement was performed in the 3D reconstruction.

### 2.5. 3D Reconstruction

Three-dimensional reconstruction was performed by interpolation of the 2D segmented images. For interpolation, every unset voxel was examined. If there were 15 voxels set in its 3 × 3 × 10 voxel neighborhood, the examined voxel was also set. Looking 10 voxels in z-direction enables the bridging of larger gaps resulting from missing image information. To close discontinued vessels resulting from inaccuracies in registration or segmentation, for every voxel its 3D neighborhood of voxels was checked again. This neighborhood is defined as a 3 × 3 × 3 voxel neighborhood around the examined voxel in the original stack. If there were less than 10 voxels identified as vessel in the original voxel neighborhood, the voxel was discarded. Both the size of the neighborhood and the value of 10 voxels are empirical values.

To achieve a smoother and in this way more natural look of vessel structures, a Gaussian filter in 3D was applied. This was followed by thresholding to receive binary images again. The smoothing also led to a more robust algorithm as remaining discontinuities in a vessel’s trajectory were eliminated.

The reconstructed 3D vessel network was qualitatively evaluated by an experienced radiologist.

## 3. Results and Discussion

In this work, we developed an algorithm to reconstruct a 3D vascular network from histological slices. The algorithm consists of a two-step registration based on the ImproveCWR algorithm by Wang et al. [25,26] (see Section 2.3), a five-step segmentation (see Section 2.4), and a 3D reconstruction (see Section 2.5).

The computing time for the registration, segmentation, and reconstruction of 169 consecutive slices of an example pancreatic ductal adenocarcinoma is shown in Table 1.

During the registration, one computational node was allocated, which then used all 48 cores, and 187 GB of main memory was allocated. For the segmentation, five computational nodes were allocated, using in total 158 cores and 750 GB of main memory. For the reconstruction, one computational node was allocated using one core and 4 GB of main memory. The total computation time of 197 min shows the benefits of the parallelization of the registration steps. If the registration were to be computed linearly on a single CPU, the runtime could be approximated to last over 66 days for the 169 slices. The total of 197 min is therefore within a reasonable time frame in a research context for the development of reliable in vitro models. This would be, however, not appropriate for, as an example, tissue analysis in a clinical setting, in which decisions are time critical.

**Figure 6:**
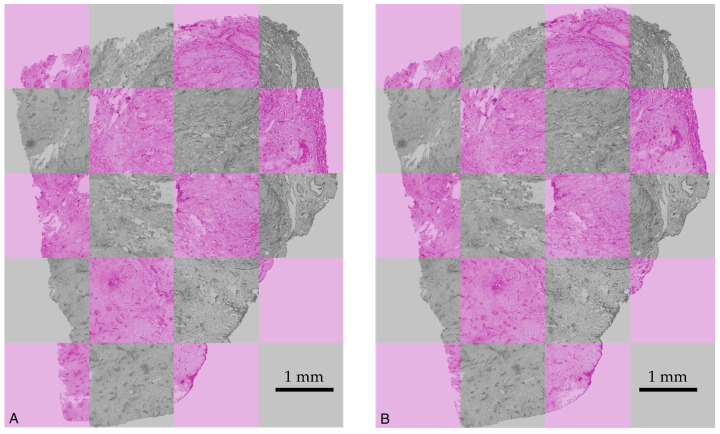
Two superimposed images of slices displayed in grey and in pink, respectively: (**A**) without registration and (**B**) after registration. In (**A**), a clear mismatch between the outlines of the slices displayed in grey and in pink is observed. In (**B**), the outlines of the slices coincide.

Figure 6 shows unregistered and registered images, demonstrating the performance of the registration algorithm.

Due to manual handling of the slices for sample preparation and digitization, image artifacts were created. Therefore, registration of the digitalized slides was necessary. Table 2 summarizes registration methods reported in the literature and their applicability for high-resolution histologic slices of tumors. For registration, physical markers can be used, which are applied to the tissue before slicing [36]. This technique has the disadvantage of damaging the tissue, which can no longer be evaluated at the position of the markers. To overcome this problem, Schwier et al. [37] introduced a method in which vessels are used as markers to register the slices as they are re-occurring features on every slice. Another approach is the scale-invariant feature transform (SIFT) by Lowe [38], which extracts distinctive areas, called features, from unregistered images that can be matched to one another. This SIFT algorithm and the similar SURF algorithm [39] are used in a couple of other registration approaches [40,41,42,43,44,45,46,47]. However, feature extraction alone in histologic images of a tumor is not sufficient because of the highly repetitive patterns that are not sufficiently distinctive. Kugler et al. [48,49] also applied the extraction of features technique using normalized cross-correlation and smoothening procedures. Rigid image registration algorithms [50,51] are frequently used, enabling the rotation, displacement, and scaling of digitalized slices. However, these algorithms are not sufficient for the registration of typical histological slices, which show further damages gained during preparation (deformations, fissures, etc.). Consequently, most existing registration methods show shortcomings in registering high-resolution images of tumor slices as they cannot handle extensive damage of tissue such as shearing [40,51,52], ruptures [53], or highly repetitive patterns [38,39,41,45,47,54,55]. Although the images of the example tumor investigated in this work consist of highly repetitive patterns, which limits exclusively feature-based approaches such as SIFT and SURF [38,39,40,41,44,47,55,56], the algorithm used is suitable to register whole slide images. This is due to the combination of area- and feature-based registration. Furthermore, it does not rely on previously segmented images [54,57].

**Table 2:** Registration methods reported in the literature, application field, and limitations of the method.

Focused Methods	Reconstruction-Related Application	Limitations
Fiducial markers for point matching		
Rigid registration [36]	Protocol for inducing fiducial markers	Markers locally destroy tissue and vessels
Affine registration [58]	3D reconstruction specifically for muscle fibers	
Feature extraction and matching methods		
Finding scale-invariant feature transformation SIFT/SURF: local feature descriptors [38,39,40,41,44]	Detection and matching of distinctive features invariant to the transformation	Unsuitable for highly repetitive patterns of histologic samples
Object tracing using SIFT [47]	Registration for images with high deformation, artifacts, and missing tissue, incorporating quality assurance	
Point matching improving RANSAC [56]	Faster and more robust point matching of features	
Non-rigid feature matching [55]	Matching and handling large deformations	
Rigid registration methods		
Sparse-feature-based registration [42]	Handling registration of objects with different orders of magnitudes in structure size	Rigid registration is not sufficient for samples deformed by shearing
Rigid registration [51]	Co-registering re-stained histological images	
Trajectory tracing [49]	Registration and reconstruction of 2D histological images with different staining	Mainly captures vessels along z-axis
Non-rigid registration methods		
Rigid and elastic registration [57]	Automatic sectioning, segmentation, registration, 3D reconstruction	Registration of already segmented images
Intensity-based registration, curvature flow [54]	Smoothed 3D reconstruction of distinct object boundaries	
Feature- and area-based registration [25]	Different image types including highly repetitive histological images	Computationally expensive transformations
B-spline [59]	3D reconstruction preserving tissue microstructures	Not capable of handling large distortions
Affine registration, mutual information (MI), matrix exponential neural network (MINE) [53]	Unsupervised registration for mono- and multi-modal images	
Registering multi-resolution scales for ROIs [45]	Registration of histological images dealing with major artifacts	No whole-section registration
Intensity-based stochastic gradient descent method (SGDM), region-based convolutional neural networks [60]	Registration, segmentation, and reconstruction of multi-modal histological images, focused on nerval specimen	Unsuitable for highly repetitive patterns of histologic samples
Non-automatic methods		
Rigid registration [50]	Manual rigid registration	Unfeasible for big data
Manual segmentation, rigid registration, mesh generation [52]	Direct 3D mesh generation from images	

The results of the validation (see Section 2.3) are shown in Table 3. The quality of the presented method ranks in the middle of the earlier published methods for the complete set of images of the ANHIR challenge and for images similar to those presented in this work at the very front [30].

It can be concluded that the results referring to the investigation of the complete set of images are in the middle of the bulk of those published in the ANHIR challenge [30] or compared to the current leaderboard [61]. However, for the image subset, the results rank within the top ranks published. Parallelization of different steps in the image registration (s. Figure 3) resulted in time-efficient computation (cf. Table 1), making the algorithm suitable for the investigation of a large number of images.

**Figure 7:**
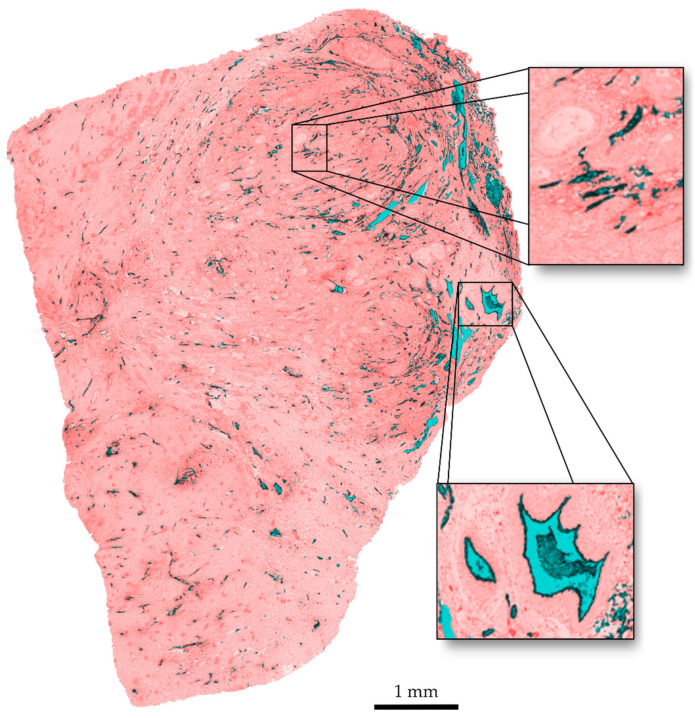
Overlay of original image and segmentation. Vessel interiors are marked in blue, vessel contours in black, discarded tissue in red.

Regarding the visual quality of the segmentation, the results are presented as an overlay of the original slice and the segmented areas in Figure 7. It can be observed that clustered vessels occur in areas with a high structure density. The delineation of individual structures with a very small space of a few pixels between them gets lost in the dilation step. This is a limitation of the algorithm. A delineation of single vessels within these clusters could be achieved for images with a higher resolution. However, this leads to an increase in computation time.

To develop a 3D vascular network from registered images of slices, vessels need to be segmented from the surrounding tissue. With regard to segmentation, for some of the published works, commercial software was used that is not available anymore [50,62] Other approaches were specifically developed for certain tissue types, such as fascicles [60], and are not applicable to histologic images of tumor tissue. Most segmentation algorithms are designed to delineate cancerous tissue regions from non-cancerous surroundings [63,64,65,66]. These algorithms identify cancerous regions by analysis of misshaped structures, color variations in staining, or different textures within the image. Recent approaches to segment histologic images use standard computer vision techniques such as color-based segmentation with thresholding [67,68] or Markov random fields [69], pattern recognition [65], or clustering such as k-means or mean-shift [70,71,72]. Histological images usually show very high resolutions at large image sizes due to their high level of detail, which makes the above-mentioned approaches very expensive in terms of computing time [70]. With the developments in computational power, the utilization of machine learning in medical image analysis has experienced a huge increase. Many approaches to identify cancerous regions have been developed and have greatly improved the field of medical image analysis [73,74,75,76,77,78,79]. However, identifying cancerous regions is a completely different task compared to delineating small structures such as capillaries in a tumor. The main differences refer to the level of detail of highly repetitive small features that need to be delineated.

Reyes-Aldasoro et al. [62] developed an algorithm for automated segmentation of vascular structures from histologic images. A region-growing algorithm detects vascular structures that are then—using characteristics of continuous vessels—joined, separated, or closed to align with a certain definition of a realistic pathway for vessels. Schwier et al. [80] used the software MeVisLab [81] to reconstruct a whole vessel tree from murine liver sections. The reported procedure combines color thresholds and object-based analysis to include spatial information and assumed characteristics of vessel trajectories such as continuity. A broad segmentation approach is Trainable Weka Segmentation [82]. It uses machine learning to distinguish between different areas and must manually be trained by a user. As the method presented by Schwier et al. [80] shows promising results for the segmentation of consecutive tissue slices, we tested this method with our images and compared it to the method described above. The results are described in the Supplementary Materials S1, Table S1.

Another approach for segmenting vascular structures is vesselness functions [83,84,85]. These functions enhance the appearance of blood vessels in medical images [86,87] and use a vesselness measure to obtain probability masks that state for each pixel if it is likely to belong to a vessel or not. One of the primary challenges here would be the heterogeneity in staining intensities and colors within the histological dataset. Vesselness functions are typically designed to detect structures with distinct intensity or contrast characteristics [83,88], which are likely to be obscured by the irregular and heterogeneous staining. As a result, these functions would exhibit inconsistent and unreliable responses, making the accurate identification of vessels difficult. Additionally, the arbitrary shapes of vessels within the tumor slices are challenging. Vesselness functions rely on detecting tubular or vessel-like shapes [87,88]. In the present data, vessels often exhibited irregular geometries, which do not conform to typical vessel shapes. Consequently, the vesselness functions are likely to fail to effectively capture these diverse structures.

The comparison of the resulting segmentation showed that for the images used in this work, our segmentation is more robust to distortions such as ruptures and tissue located in vessels (see Supplementary Materials S1, Table S1).

**Figure 8:**
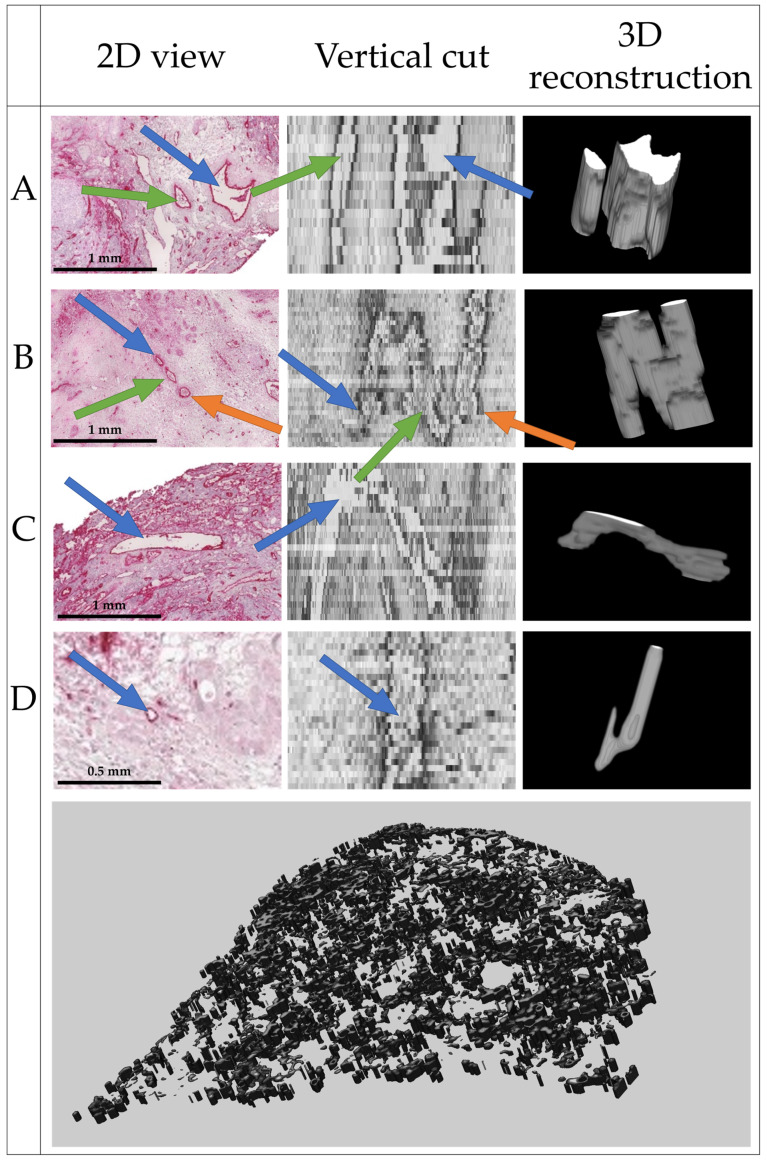
Final 3D reconstruction of the 169 slices with close-up views on four distinct vessels, where (**A**) shows two parallel vessels; (**B**) shows two bifurcations, one join and one split of two vessels each; (**C**) shows a vessel perpendicular to the image plane; and (**D**) shows a very small vessel. Within one row, arrows of the same color point to the same structures.

The complete reconstruction of the vascular network from 169 histologic slices is shown in Figure 8. Single vessels and bifurcations are clearly visible.

As no ground truth is available, the quality of the segmentation and 3D reconstruction was evaluated visually by an experienced radiologist (see Acknowledgements). Based on the results shown in Figure 8, the radiologist estimated a realistic delineation of vessels parallel and perpendicular to the image plane of the histologic slices. Some artifacts were present, but the general structure of the vessels was well represented if the vessels were visible in the histopathological source images.

A validation of the continuity of the vessels was not possible with the available dataset. As the histologic slices have a thickness of 2.5 µm, the reconstruction of the 169 slices represents a vessel network with a thickness of 422.5 µm. For an assessment of vessel continuity, a larger dataset is necessary. Therefore, the evaluation of vessel continuity is subject to future research.

Reconstruction of 3D structures based on histological images has been performed before for different medical applications: Feuerstein et al. [89] used Markov random fields with deformable stack registration. Fonyad et al. [90] reconstructed a coronary model of mice using the Voloom software (former µCore by microDimensions GmbH, Munich, Germany). Onozato et al. [91] reconstructed adenocarcinomas of the lung using a former version of the CaseViewer software (3DHistech Ltd., Budapest, Hungary). Another predecessor of CaseViewer was used by Wu et al. [92] to reconstruct colorectal biopsies. Tovbis et al. [60] additionally segmented the fascicles in the reconstructed tissue. The approach presented here focuses on the reconstruction of capillaries on a much smaller scale than previous works.

## 4. Conclusions

With this work, we developed an algorithm that enables the reconstruction of a 3D vascular network of a human tumor based on histologic images. The algorithm consists of a two-step registration approach, feature- and area-based registration, and is suitable for the investigation of a large number of high-resolution images. It was validated with the ANHIR dataset and competes with the top ranks for similar tissue types as used in this work. It further uses a five-step segmentation, which separately delineates vessel interiors and contours, and fuses this information with that from consecutive images, to extract the vessels from the surrounding tissue. The reconstruction algorithm refines the registration and segmentation results to achieve a natural-looking model of a vascular network. The resulting 3D model was qualitatively evaluated by an experienced radiologist and considered as promising.

## Figures and Tables

**Table 1:** Computation time of the different tasks to reconstruct a 3D vascular network from histologic slices.

Task	Time for Completion (in Minutes)
registration (step 1): calculation of transformation of each slice to its unregistered predecessor	11.93
registration (step 2): application of all predeceasing transformations to each slice	58.78
complete registration (step 1 + step 2)	70.71
segmentation	72.28
reconstruction	54.38
total computation time	197.37

**Table 3:** Results of the registration validation. The registration error (*r*TRE) and the average robustness *R*_i_ are calculated as described in Section 2.3 and compared to results from literature.

	Complete Set of Images	Subset of Similar Images	Range of Top 10 Algorithms in ANHIR Leaderboard [61]
average median rTRE	0.0044	0.002163	0.00067–0.00250
average robustness *R_i_*	0.85	0.986152	0.99276–0.88992

## Data Availability

Data are available in a pseudonymized manner upon request due to ethical restrictions (patient confidentially). Therefore, the data are not available publicly.

## Supplementary Materials

The following are available online at https://www.mdpi.com/article/10.3390/cancers15225352/s1, Chapter S1: Comparison to alternative segmentation algorithm, Table S1: Comparison of the segmentation algorithms. Reference [80] is cited in the Supplementary Materials.

## References

[1] Chen Y., Ali M., Shi S., Cheang U.K. (2019). Biosensing-by-Learning Direct Targeting Strategy for Enhanced Tumor Sensitization. IEEE Trans. Nanobioscience.

[2] Haun J.B., Yoon T.-J., Lee H., Weissleder R. (2010). Magnetic nanoparticle biosensors. Wiley Interdiscip. Rev. Nanomed. Nanobiotechnol..

[3] Bhatia S.N., Ingber D.E. (2014). Microfluidic organs-on-chips. Nat. Biotechnol..

[4] Koyilot M.C., Natarajan P., Hunt C.R., Sivarajkumar S., Roy R., Joglekar S., Pandita S., Tong C.W., Marakkar S., Subramanian L. (2022). Breakthroughs and Applications of Organ-on-a-Chip Technology. Cells.

[5] Bongio M., Lopa S., Gilardi M., Bersini S., Moretti M. (2016). A 3D vascularized bone remodeling model combining osteoblasts and osteoclasts in a CaP nanoparticle-enriched matrix. Nanomedicine.

[6] Unger C., Kramer N., Walzl A., Scherzer M., Hengstschläger M., Dolznig H. (2014). Modeling human carcinomas: Physiologically relevant 3D models to improve anti-cancer drug development. Adv. Drug Deliv. Rev..

[7] Abd Elrahman A.A., Mansour F.R. (2019). Targeted magnetic iron oxide nanoparticles: Preparation, functionalization and biomedical application: Preparation, functionalization and biomedical application. J. Drug Deliv. Sci. Technol..

[8] Dadfar S.M., Roemhild K., Drude N.I., von Stillfried S., Knüchel R., Kiessling F., Lammers T. (2019). Iron oxide nanoparticles: Diagnostic, therapeutic and theranostic applications. Adv. Drug Deliv. Rev..

[9] Chawla A., Ferrone C.R. (2019). Neoadjuvant Therapy for Resectable Pancreatic Cancer: An Evolving Paradigm Shift. Front. Oncol..

[10] Roeth A.A., Slabu I., Baumann M., Alizai P.H., Schmeding M., Guentherodt G., Schmitz-Rode T., Neumann U.P. (2017). Establishment of a biophysical model to optimize endoscopic targeting of magnetic nanoparticles for cancer treatment. Int. J. Nanomed..

[11] Lindemann M.C., Luttke T., Nottrodt N., Schmitz-Rode T., Slabu I. (2021). FEM based simulation of magnetic drug targeting in a multibranched vessel model. Comput. Methods Programs Biomed..

[12] Singh M., Ma R., Zhu L. (2021). Quantitative evaluation of effects of coupled temperature elevation, thermal damage, and enlarged porosity on nanoparticle migration in tumors during magnetic nanoparticle hyperthermia. Int. Commun. Heat Mass Transf..

[13] Singh M., Singh T., Soni S. (2021). Pre-operative Assessment of Ablation Margins for Variable Blood Perfusion Metrics in a Magnetic Resonance Imaging Based Complex Breast Tumour Anatomy: Simulation Paradigms in Thermal Therapies. Comput. Methods Programs Biomed..

[14] Van der Meer A.D., van den Berg A. (2012). Organs-on-chips: Breaking the in vitro impasse. Integr. Biol..

[15] Russell W., Burch R.L. (1959). The Principles of Humane Experimental Technique.

[16] Roeth A.A., Garretson I., Beltz M., Herbold T., Schulze-Hagen M., Quaisser S., Georgens A., Reith D., Slabu I., Klink C.D. (2021). 3D-Printed Replica and Porcine Explants for Pre-Clinical Optimization of Endoscopic Tumor Treatment by Magnetic Targeting. Cancers.

[17] Wang P., Sun Y., Shi X., Shen H., Ning H., Liu H. (2021). 3D printing of tissue engineering scaffolds: A focus on vascular regeneration. Bio-Des. Manuf..

[18] Szafron J.M., Ramachandra A.B., Breuer C.K., Marsden A.L., Humphrey J.D. (2019). Optimization of Tissue-Engineered Vascular Graft Design Using Computational Modeling. Tissue Eng. Part C Methods.

[19] Tu S., Hu F., Cai W., Xiao L., Zhang L., Zheng H., Jiang Q., Chen L. (2017). Visualizing polymeric bioresorbable scaffolds with three-dimensional image reconstruction using contrast-enhanced micro-computed tomography. Int. J. Cardiovasc. Imaging.

[20] Ghaghada K.B., Sato A.F., Starosolski Z.A., Berg J., Vail D.M. (2016). Computed Tomography Imaging of Solid Tumors Using a Liposomal-Iodine Contrast Agent in Companion Dogs with Naturally Occurring Cancer. PLoS ONE.

[21] Ghani M.U., Zhou Z., Ren L., Li Y., Zheng B., Yang K., Liu H. (2016). Investigation of spatial resolution characteristics of an in vivo micro computed tomography system. Nucl. Instrum. Methods Phys. Res. A.

[22] McDonald D.M., Choyke P.L. (2003). Imaging of angiogenesis: From microscope to clinic. Nat. Med..

[23] Pena A.-M., Chen X., Pence I.J., Bornschlögl T., Jeong S., Grégoire S., Luengo G.S., Hallegot P., Obeidy P., Feizpour A. (2020). Imaging and quantifying drug delivery in skin—Part 2: Fluorescence andvibrational spectroscopic imaging methods. Adv. Drug Deliv. Rev..

[24] Schindelin J., Arganda-Carreras I., Frise E., Kaynig V., Longair M., Pietzsch T., Preibisch S., Rueden C., Saalfeld S., Schmid B. (2012). Fiji: An open-source platform for biological-image analysis. Nat. Methods.

[25] Wang C.-W., Chen H.-C. (2013). Improved image alignment method in application to X-ray images and biological images. Bioinformatics.

[26] Wang C.-W., Ka S.-M., Chen A. (2014). Robust image registration of biological microscopic images. Sci. Rep..

[27] Arganda-Carreras I., Sorzano C.O.S., Marabini R., Carazo J.M., Ortiz-De-Solorzano C., Kybic J., Hutchison D., Kanade T., Kittler J., Kleinberg J.M., Mattern F., Mitchell J.C., Naor M., Nierstrasz O., Pandu Rangan C. (2006). Consistent and Elastic Registration of Histological Sections Using Vector-Spline Regularization. Computer Vision Approaches to Medical Image Analysis.

[28] Sorzano C.O.S., Thévenaz P., Unser M. (2005). Elastic registration of biological images using vector-spline regularization. IEEE Trans. Biomed. Eng..

[29] Borovec J., Munoz-Barrutia A., Kybic J. Benchmarking of Image Registration Methods for Differently Stained Histological Slides. Proceedings of the 2018 25th IEEE International Conference on Image Processing (ICIP).

[30] Borovec J., Kybic J., Arganda-Carreras I., Sorokin D.V., Bueno G., Khvostikov A.V., Bakas S., Chang E.I.-C., Heldmann S., Kartasalo K. (2020). ANHIR: Automatic Non-Rigid Histological Image Registration Challenge. IEEE Trans. Med. Imaging.

[31] Zuiderveld K., Heckbert P.S. (1994). Contrast limited adaptive histogram equilization. Graphics Gems IV.

[32] Otsu N. (1979). A Threshold Selection Method from Gray-Level Histograms. IEEE Trans. Syst., Man Cybern..

[33] Gonzalez R.C., Woods R.E., Eddins S.L. (2004). Digital Image Processing Using MATLAB.

[34] Reddy P.R., Amarnadh V., Bhaskar M. (2012). Evaluation of Stopping Criterion in Contour Tracing Algorithms. Int. J. Comput. Sci. Inf. Technol..

[35] Haralick R.M., Shapiro L.G. (1993). Computer and Robot Vision, 2.

[36] Hughes C., Rouvière O., Mege-Lechevallier F., Souchon R., Prost R. (2013). Robust alignment of prostate histology slices with quantified accuracy. IEEE Trans. Biomed. Eng..

[37] Schwier M., Böhler T., Hahn H.K., Dahmen U., Dirsch O. (2013). Registration of histological whole slide images guided by vessel structures. J. Pathol. Inform..

[38] Lowe D.G. (2004). Distinctive Image Features from Scale-Invariant Keypoints. Int. J. Comput. Vis..

[39] Bay H., Ess A., Tuytelaars T., van Gool L. (2008). Speeded-Up Robust Features (SURF). Comput. Vis. Image Underst..

[40] Kupfer B., Netanyahu N.S., Shimshoni I. (2015). An Efficient SIFT-Based Mode-Seeking Algorithm for Sub-Pixel Registration of Remotely Sensed Images. IEEE Geosci. Remote Sens. Lett..

[41] Liu Q., Zhao G., Deng J., Xue Q., Hou W., He Y. Image Registration Algorithm for Sequence Pathology Slices of Pulmonary Nodule. Proceedings of the 2019 8th International Symposium on Next Generation Electronics (ISNE).

[42] Lobachev O., Ulrich C., Steiniger B.S., Wilhelmi V., Stachniss V., Guthe M. (2017). Feature-based multi-resolution registration of immunostained serial sections. Med. Image Anal..

[43] Saalfeld S., Fetter R., Cardona A., Tomancak P. (2012). Elastic volume reconstruction from series of ultra-thin microscopy sections. Nat. Methods.

[44] Hermann J., Brehmer K., Jankowski V., Lellig M., Hohl M., Mahfoud F., Speer T., Schunk S.J., Tschernig T., Thiele H. (2020). Registration of Image Modalities for Analyses of Tissue Samples Using 3D Image Modelling. Proteom. Clin. Appl..

[45] Paknezhad M., Loh S.Y.M., Choudhury Y., Koh V.K.C., Yong T.T.K., Tan H.S., Kanesvaran R., Tan P.H., Peng J.Y.S., Yu W. (2020). Regional registration of whole slide image stacks containing major histological artifacts. BMC Bioinform..

[46] Zhang J., Li Z., Yu Q. Point-Based Registration for Multi-stained Histology Images. Proceedings of the 2020 IEEE 5th International Conference on Image, Vision and Computing (ICIVC).

[47] Deng R., Yang H., Jha A., Lu Y., Chu P., Fogo A.B., Huo Y. (2021). Map3D: Registration-Based Multi-Object Tracking on 3D Serial Whole Slide Images. IEEE Trans. Med. Imaging.

[48] Kugler M., Goto Y., Kawamura N., Kobayashi H., Yokota T., Iwamoto C., Ohuchida K., Hashizume M., Hontani H., Stoyanov D., Taylor Z., Ciompi F., Xu Y., Martel A., Maier-Hein L., Rajpoot N., van der Laak J., Veta M., McKenna S. (2018). Accurate 3D Reconstruction of a Whole Pancreatic Cancer Tumor from Pathology Images with Different Stains. Computational Pathology and Ophthalmic Medical Image Analysis: First International Workshop, COMPAY 2018, and 5th International Workshop, OMIA 2018, held in Conjunction with MICCAI 2018, Granada, Spain, 16–20 September 2018: Proceedings.

[49] Kugler M., Goto Y., Tamura Y., Kawamura N., Kobayashi H., Yokota T., Iwamoto C., Ohuchida K., Hashizume M., Shimizu A. (2019). Robust 3D image reconstruction of pancreatic cancer tumors from histopathological images with different stains and its quantitative performance evaluation. Int. J. Comput. Assist. Radiol. Surg..

[50] Paish E.C., Green A.R., Rakha E.A., Macmillan R.D., Maddison J.R., Ellis I.O. (2009). Three-dimensional reconstruction of sentinel lymph nodes with metastatic breast cancer indicates three distinct patterns of tumour growth. J. Clin. Pathol..

[51] Jiang J., Larson N.B., Prodduturi N., Flotte T.J., Hart S.N. (2019). Robust hierarchical density estimation and regression for re-stained histological whole slide image co-registration. PLoS ONE.

[52] Niemann A., Weigand S., Hoffmann T., Skalej M., Tulamo R., Preim B., Saalfeld S. (2020). Interactive exploration of a 3D intracranial aneurysm wall model extracted from histologic slices. Int. J. Comput. Assist. Radiol. Surg..

[53] Nan A., Tennant M., Rubin U., Ray N. DRMIME: Differentiable Mutual Information and Matrix Exponential for Multi-Resolution Image Registration. Proceedings of the Third Conference on Medical Imaging with Deep Learning.

[54] Cifor A., Bai L., Pitiot A. (2011). Smoothness-guided 3-D reconstruction of 2-D histological images. Neuroimage.

[55] Cai N., Chen H., Li Y., Peng Y., Li J., Li X. (2019). Reducing non-realistic deformations in registration using precise and reliable landmark correspondences. Comput. Biol. Med..

[56] Wu Y., Ma W., Gong M., Su L., Jiao L. (2015). A Novel Point-Matching Algorithm Based on Fast Sample Consensus for Image Registration. IEEE Geosci. Remote Sens. Lett..

[57] Arganda-Carreras I., Fernández-González R., Muñoz-Barrutia A., Ortiz-De-Solorzano C. (2010). 3D reconstruction of histological sections: Application to mammary gland tissue. Microsc. Res. Tech..

[58] Kuravi R., Leichsenring K., Böl M., Ehret A.E. (2020). 3D finite element models from serial section histology of skeletal muscle tissue —The role of micro-architecture on mechanical behaviour. J. Mech. Behav. Biomed. Mater..

[59] Liu J., Wu X., Xu C., Ma M., Zhao J., Li M., Yu Q., Hao X., Wang G., Wei B. (2020). A Novel Method for Observing Tumor Margin in Hepatoblastoma Based on Microstructure 3D Reconstruction. Fetal Pediatr. Pathol..

[60] Tovbis D., Agur A., Mogk J.P.M., Zariffa J. (2020). Automatic three-dimensional reconstruction of fascicles in peripheral nerves from histological images. PLoS ONE.

[61] Borovec J., Kybic J., Barrutia A.M. Leaderboard—Grand Challenge. https://anhir.grand-challenge.org/evaluation/challenge/leaderboard/.

[62] Reyes-Aldasoro C.C., Williams L.J., Akerman S., Kanthou C., Tozer G.M. (2011). An automatic algorithm for the segmentation and morphological analysis of microvessels in immunostained histological tumour sections. J. Microsc..

[63] Li C., Chen H., Li X., Xu N., Hu Z., Xue D., Qi S., Ma H., Zhang L., Sun H. (2020). A review for cervical histopathology image analysis using machine vision approaches. Artif. Intell. Rev..

[64] Azevedo Tosta T.A., Neves L.A., do Nascimento M.Z. (2017). Segmentation methods of H&E-stained histological images of lymphoma: A review. Inform. Med. Unlocked.

[65] Tabesh A., Teverovskiy M., Pang H.-Y., Kumar V.P., Verbel D., Kotsianti A., Saidi O. (2007). Multifeature prostate cancer diagnosis and Gleason grading of histological images. IEEE Trans. Med. Imaging.

[66] Gurcan M.N., Boucheron L.E., Can A., Madabhushi A., Rajpoot N.M., Yener B. (2009). Histopathological image analysis: A review. IEEE Rev. Biomed. Eng..

[67] Goddard J.C., Sutton C.D., Furness P.N., Kockelbergh R.C., O’Byrne K.J. (2002). A computer image analysis system for microvessel density measurement in solid tumours. Angiogenesis.

[68] Steiniger B., Bette M., Schwarzbach H. (2011). The open microcirculation in human spleens: A three-dimensional approach. J. Histochem. Cytochem..

[69] Meas-Yedid V., Tilie S., Olivo-Marin J.-C. Color image segmentation based on Markov random field clustering for histological image analysis. Proceedings of the 16th International Conference on Pattern Recognition.

[70] Wu G., Zhao X., Luo S., Shi H. (2015). Histological image segmentation using fast mean shift clustering method. Biomed. Eng. Online.

[71] Yang L., Meer P., Foran D.J. (2005). Unsupervised segmentation based on robust estimation and color active contour models. IEEE Trans. Inf. Technol. Biomed..

[72] He L., Long L.R., Antani S., Thoma G. (2011). Distribution fitting-based pixel labeling for histology image segmentation. Med. Imaging 2011 Comput.-Aided Diagn..

[73] Xu Y., Zhu J.-Y., Chang E.I.-C., Lai M., Tu Z. (2014). Weakly supervised histopathology cancer image segmentation and classification. Med. Image Anal..

[74] Rodríguez R. (2003). Blood Vessel Segmentation via Neural Network in Histological Images. J. Intell. Robot. Syst..

[75] Kothari S., Phan J.H., Moffitt R.A., Stokes T.H., Hassberger S.E., Chaudry Q., Young A.N., Wang M.D. (2011). Automatic batch-invariant color segmentation of histological cancer images. Proc. IEEE Int. Symp. Biomed. Imaging.

[76] Jia Z., Huang X., Chang E.I.-C., Xu Y. (2017). Constrained Deep Weak Supervision for Histopathology Image Segmentation. IEEE Trans. Med. Imaging.

[77] Xu G., Song Z., Sun Z., Ku C., Yang Z., Liu C., Wang S., Ma J., Xu W. CAMEL: A Weakly Supervised Learning Framework for Histopathology Image Segmentation. Proceedings of the 2019 IEEE/CVF International Conference on Computer Vision (ICCV).

[78] Sun C., Li C., Zhang J., Rahaman M.M., Ai S., Chen H., Kulwa F., Li Y., Li X., Jiang T. (2020). Gastric histopathology image segmentation using a hierarchical conditional random field. Biocybern. Biomed. Eng..

[79] Janssens T., Antanas L., Derde S., Vanhorebeek I., van den Berghe G., Güiza Grandas F. (2013). Charisma: An integrated approach to automatic H&E-stained skeletal muscle cell segmentation using supervised learning and novel robust clump splitting. Med. Image Anal..

[80] Schwier M., Hahn H.K., Dahmen U., Dirsch O., Gurcan M.N., Madabhushi A. (2013). Reconstruction of vessel structures from serial whole slide sections of murine liver samples. Proceedings of the SPIE Medical Imaging.

[81] Ritter F., Boskamp T., Homeyer A., Laue H., Schwier M., Link F., Peitgen H.-O. (2011). Medical image analysis. IEEE Pulse.

[82] Arganda-Carreras I., Kaynig V., Rueden C., Eliceiri K.W., Schindelin J., Cardona A., Sebastian Seung H. (2017). Trainable Weka Segmentation: A machine learning tool for microscopy pixel classification. Bioinformatics.

[83] Drechsler K., Oyarzun Laura C. Comparison of vesselness functions for multiscale analysis of the liver vasculature. Proceedings of the 2010 10th IEEE International Conference on Information Technology and Applications in Biomedicine (ITAB 2010).

[84] Tetteh G., Efremov V., Forkert N.D., Schneider M., Kirschke J., Weber B., Zimmer C., Piraud M., Menze B.H. (2020). DeepVesselNet: Vessel Segmentation, Centerline Prediction, and Bifurcation Detection in 3-D Angiographic Volumes. Front. Neurosci..

[85] Woźniak T., Strzelecki M., Majos A., Stefańczyk L. (2017). 3D vascular tree segmentation using a multiscale vesselness function and a level set approach. Biocybern. Biomed. Eng..

[86] Survarachakan S., Pelanis E., Khan Z.A., Kumar R.P., Edwin B., Lindseth F. (2021). Effects of Enhancement on Deep Learning Based Hepatic Vessel Segmentation. Electronics.

[87] Frangi A.F., Niessen W.J., Vincken K.L., Viergever M.A., Wells W.M., Colchester A., Delp S. (1998). Multiscale vessel enhancement filtering. Medical Image Computing and Computer-Assisted Interventation—MICCAI’98.

[88] Lamy J., Merveille O., Kerautret B., Passat N., Vacavant A. Vesselness Filters: A Survey with Benchmarks Applied to Liver Imaging. Proceedings of the 2020 25th International Conference on Pattern Recognition (ICPR).

[89] Feuerstein M., Heibel H., Gardiazabal J., Navab N., Groher M. (2011). Reconstruction of 3-D histology images by simultaneous deformable registration. Med. Image Comput. Comput. Assist. Interv..

[90] Fónyad L., Shinoda K., Farkash E.A., Groher M., Sebastian D.P., Szász A.M., Colvin R.B., Yagi Y. (2015). 3-dimensional digital reconstruction of the murine coronary system for the evaluation of chronic allograft vasculopathy. Diagn. Pathol..

[91] Onozato M.L., Klepeis V.E., Yagi Y., Mino-Kenudson M. (2012). A role of three-dimensional (3D) reconstruction in the classification of lung adenocarcinoma. Stud. Health Technol. Inform..

[92] Wu M.L., Varga V.S., Kamaras V., Ficsor L., Tagscherer A., Tulassay Z., Molnar B. (2005). Three-dimensional virtual microscopy of colorectal biopsies. Arch. Pathol. Lab. Med..

