# Supplementary material for: Towards Realistic 3D Models of Tumor Vascular Networks
Source: Cancers (Basel). 2023 Nov 9;15(22):5352. doi: 10.3390/cancers15225352 (PMC10670125; doi:10.3390/cancers15225352)
Supplement: Supplementary file 1 [file cancers-15-05352-s001.zip › cancers-2607496-supplementary.pdf]

cancers MDPI

Supplementary Data

Towards a realistic 3D-model of a tumor vascular network

Max C. Lindemann [1], Lukas Glänzer [1], Anjali A. Roeth [2], Thomas Schmitz-Rode [1] and Ioana Slabu [1,*]

[1] Institute of Applied Medical Engineering, Helmholtz Institute, Medical Faculty, RWTH Aachen University, Pauwelsstraße 20, 52074 Aachen, Germany
[2] Department of Visceral and Transplantation Surgery, University Hospital RWTH Aachen, Pauwelsstrasse 30, 52074 Aachen, Germany
[3] Department of Surgery, Maastricht University, P. Debyelaan 25, 6229 HX Maastricht, The Netherlands
* Correspondence: slabu@ame.rwth-aachen.de; Tel.: +49 241 80 89102 (I.S.)
# These authors contributed equally to this work.

S1. Comparison to alternative segmentation algorithm

The algorithm developed by Schwier et. al. [1] showed promising results for segmenting vascular structures and constructing a vessel tree. Their work, however, is based on images with a resolution of (1056 x 1025) px^2 with a pixel spacing of 14.5 µm. Additionally, Schwier et. al. based their work on murine liver samples. The vascular structures show different characteristics. Mainly, they are better distinguishable from the image background. Therefore, a combination of contrast enhancement and color threshold is sufficient for segmenting these structures. However, vascular structures in the images used in this work are often not distinguishable from the background by just using a threshold. The segmentation presented in this work incorporates contrast enhancement and thresholding as well, yet, additional steps are necessary to segment the desired vessels.

Table S1 compares the segmentation algorithm developed by Schwier et. al. [1] with the method presented in this work. It shows that the algorithm presented by Schwier et. al. only detects those vessel interiors that are clearly distinguishable from their surrounding tissue (green). Structures showing an interior (blue) are hardly detected. Furthermore, some tissue is classified as vessel although it is torn tissue or tissue with little coloring. (yellow). The algorithm developed by Schwier et. al. is, therefore, limited to bigger structures, which have a visible interior area. The segmentation presented in this work detects far more structures including those, which do not have any interior area.

Table S1. Comparison of the segmentation algorithms. A histological image (A) is segmented using the method proposed by Schwier et. al. [1] (B) and using the method presented in this work (C).

Original Image	Segmentation after [1]	Segmentation proposed in this work
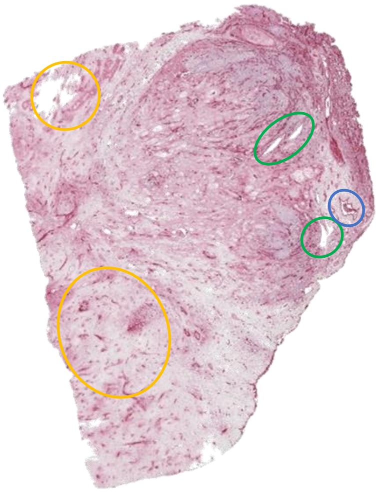 A	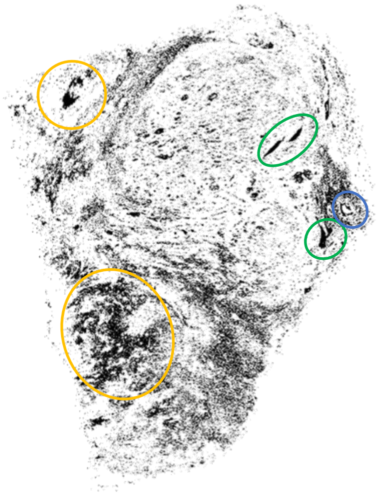 B	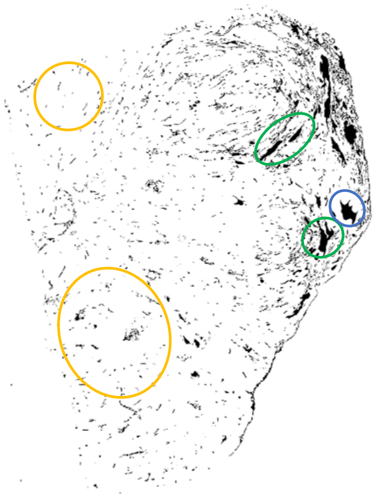 C

References

1. Schwier, M.; Hahn, H.K.; Dahmen, U.; Dirsch, O. Reconstruction of vessel structures from serial whole slide sections of murine liver samples. In . SPIE Medical Imaging, Lake Buena Vista (Orlando Area), Florida, USA, Saturday 9 February 2013; Gurcan, M.N., Madabhushi, A., Eds.; SPIE, 2013; 86760D.
